# Data-Driven Guideline Adherence in Data Representation and Compliance Measurement: Scoping Review

**DOI:** 10.2196/79937

**Published:** 2026-02-09

**Authors:** Minh Trang Hoang, Candice Donnelly, Christina Igasto, Amith Shetty, Malcolm Pradhan, Tim Shaw

**Affiliations:** 1 Biomedical Informatics and Digital Health, School of Medical Sciences, Faculty of Medicine and Health, The University of Sydney, Sydney, Australia, 61 401333970; 2 Service NSW, Sydney, Australia; 3 NSW Ministry of Health, Sydney, Australia; 4 Western Sydney Local Health District, Westmead, Australia

**Keywords:** clinical practice guidelines, best practice, guideline compliance, computer-interpretable guidelines, electronic medical records, clinical decision support

## Abstract

**Background:**

Best practice standards aim to standardize care and improve outcomes. However, variation in clinical practice exists, and not all deviations are inappropriate. Measuring adherence to best practice standards remains challenging due to limitations in representation methods and data fidelity.

**Objective:**

This scoping review aims to survey and synthesize the existing literature on the computable representation of guideline recommendations and to explore methods for detecting and quantifying deviations from best practice standards.

**Methods:**

We followed the Arksey and O’Malley framework and PRISMA-ScR (Preferred Reporting Items for Systematic Reviews and Meta-Analyses Extension for Scoping Reviews) guidelines. Five databases (Ovid Medline, EMBASE, IEEE Xplore, Web of Science, and Scopus) were searched in November 2025. Studies were included if they either (1) described a computer representation of best practice standards or (2) assessed adherence to such standards using patient data, including patient data derived from electronic medical records or event logs. Screening was done using Covidence (Veritas Health Innovation). Data were extracted on representation, clinical context, data sources, adherence metrics, and modeling techniques. A narrative synthesis was conducted to identify themes.

**Results:**

Twenty-four studies were included. Most studies were published as conference proceedings (13/24, 54%). Fourteen studies (14/24, 58%) included measurement of adherence to best practice standards. Cardiovascular conditions were the most common focus (13/24, 54%). Data sources included Health Level Seven (HL7) messages, structured electronic medical record data, event logs, and Fast Healthcare Interoperability Resources (FHIR)-transformed data. Best practice standards were formalized using Business Process Model and Notation (BPMN; 6/24, 25%), ontologies (7/24, 29%), FHIR (4/24, 17%), or hybrid approaches (4/24, 17%). The most common method for adherence measurement was rule-based alignment. Several studies incorporated weighted scoring to differentiate the severity of deviations. Process mining was used in a subset to detect sequence and timing variations. However, most models lacked contextual sensitivity and rarely incorporated patient-specific factors, such as comorbidities, patient acuity, or clinician rationale. Consequently, although deviations can be automatically identified, determining whether they were clinically warranted remained largely unresolved.

**Conclusions:**

Despite promising advances, challenges persist in computer-interpretable representation and measuring adherence in a clinically meaningful way. Current approaches predominantly assess technical alignment rather than clinical relevance and are limited by data quality and standardization, thereby limiting real-world utility. This scoping review offers an innovative contribution by synthesizing evidence from 2 separate domains—the computable representation of best practice standards and the measurement of adherence. The findings emphasize the need for context-aware, standardized modeling and integration with clinical workflows to distinguish warranted from unwarranted deviations. Such advances are essential for scalable, transparent, and real-time adherence monitoring—ultimately driving safer, patient-centered care delivery.

## Introduction

Delivering patient care that aligns with evidence-based best practice has been associated with many benefits, including increased hospital efficiency, decreased operational costs, and improved patient outcomes [1-4], especially reduced length of stay and decreased mortality rate [4]. This is achievable through best practice standards, which are typically reflected in clinical practice guidelines (CPGs). The most cited definition refers to CPGs as “systematically developed statements to assist practitioner and patient decisions about appropriate health care for specific clinical circumstances” [5]. In the literature, terms such as “protocols,” “practice policies,” “clinical algorithms,” and “clinical pathways” are sometimes used to refer to CPGs, although they differ in scope, specificity, and intended use [6]. While CPGs are narrative documents that provide overall evidence-based recommendations, clinical pathways (CPs) translate these guidelines into structured, time-sequenced clinical processes of care for a specific problem within a particular population [7]. CPs are typically developed at the institutional or regional level and are informed by international or national guidelines but tailored to local contexts [7]. Their structured, time-sequential nature may make them well-suited as benchmarks for monitoring and evaluating clinical practice.

Despite the recognized benefits and availability of CPGs and CPs, a persistent gap exists between recommended care and clinical practice delivery [8,9]. This discrepancy, often referred to as practice variations or pathway deviations, can reflect both unwarranted and warranted deviations from practice. Practice variations may indicate erroneous care resulting in adverse patient outcomes and increased health care expenses and are often referred to as unwarranted deviations. While some practice variations may be deemed warranted deviation when individualized care is provided to accommodate patient-specific needs. Distinguishing between unwarranted and warranted deviations is crucial to understanding and improving clinical practice. However, existing approaches to measuring adherence often rely on aggregated metrics or single process indicators and fail to capture the nuance and temporal complexity of clinical care.

Advancements in digital technology play a pivotal role in delivering health care and enabling systematic and data-driven monitoring of adherence to best practice standards [10]. Electronic medical records (eMRs) offer a large volume of time-stamped, structured clinical data that can be used to evaluate adherence to best practice. In addition, Clinical Decision Support Systems (CDSS) embedded within eMRs can promote adherence to best practice standards by delivering evidence-based recommendations in a variety of care aspects [11,12]. As a result, it has also led to the emergence of computer-interpretable guidelines (CIGs), which transform narrative CPGs or CPs into machine-readable, logical representations to support automated assessment of adherence.

Multiple methodologies have been developed to support the representation of best practice standards in computable formats. Guideline modeling, such as GLIF3, Asbru, SAGE, PROforma, as well as workflow and interoperability frameworks such as Business Process Model and Notation (BPMN) and Fast Healthcare Interoperability Resources (FHIR), offer different strengths in terms of expressiveness, configurability, and system integration [13-17]. In addition, Clinical Quality Language (CQL) [18], a high-level, domain-specific language used to support the creation and execution of clinical decision support rules and algorithms. However, the heterogeneity of these approaches introduces challenges in clinical implementation and usability, and cross-system interoperability [17]. Additionally, the availability of data and different interpretations of adherence measures continue to pose a challenge to the measurement of adherence to CPGs [19,20]. Nevertheless, a lack of standardized metrics and limited consideration of clinical context continue to constrain the practical utility and adoption of these methods in clinical practice.

A recent scoping review provides summaries on the role of informatics in addressing practice variations and the clinical tasks in which practice variations occur [21]. There remains a need to understand the methodologies behind the representation of best practice standards and how these representations enable the measurement of adherence to best practice. This scoping review aims to survey and synthesize the existing literature on the computable representation of guideline recommendations and to explore the extent to which these representations have been used to detect and quantify deviations from best practice. By doing so, this review highlights methodological and operational gaps in current approaches and identifies opportunities to advance adherence measurement in digital health systems.

## Methods

### Study Design and Framework

This scoping review was conducted following the methodology and guidelines by Arksey and O’Malley [22]. In line with scoping review conventions, no quality appraisal of the included studies was conducted, as the objective was to map the breadth of a diverse range of work.

### Study Protocol

The PRISMA-ScR (Preferred Reporting Items for Systematic Reviews and Meta-Analyses Extension for Scoping Reviews) [23] was used to develop our review protocol. As a recommended practice, we used a checklist when reporting results to enhance transparency and reproducibility (eg, by sharing the exact search strategy and source databases). A complete checklist is available in Checklist 1.

#### Information Sources and Search Strategy

We searched 5 databases: Ovid Medline, EMBASE, IEEE Xplore, Web of Science, and Scopus. No study registries were searched. We also did not purposefully look for any online or print source. The last search was conducted in November 2025. The search strategy was developed in consultation with an experienced university librarian, without adapting previous search strategies from other literature reviews. It included combinations of controlled vocabulary and keywords related to clinical guidelines, clinician adherence, variation, and eMRs. We did not impose any time limit on obtaining a comprehensive overview of published articles. We reported the search according to PRISMA-S (an Extension of the Preferred Reporting Items for Systematic Reviews and Meta-Analyses Statement) guidelines [24], which are available in Checklist 2. Additional articles were identified through backward searching of the reference lists in the included studies and the authors’ collection. No additional studies or data were sought by contacting authors, experts, and manufacturers.

The references were imported to Covidence (Veritas Health Innovation) [25] for deduplication. During title and abstract screening, additional duplicates and irrelevant entries were manually removed. Covidence was also used for full-text screening, data extraction, and charting.

#### Eligibility Criteria

In this review, CPGs and CPs were considered as best practice standards as they provide evidence-based actionable recommendations. Our focus was on studies conducted in tertiary care settings, where guideline implementation and adherence monitoring are most frequently studied.

To maintain a focus on comprehensive care delivery and computable representations of best practice, we included studies that either (1) described or modeled a computer representation of best practice standards, or (2) assessed adherence to such standards using patient data, such as eMR-derived patient data or patient event logs. Studies were eligible if they captured multiple (>1) guidelines or components of care in the patient pathway. Studies focused solely on a single component, such as medication adherence—while important—were excluded, as they addressed only a single aspect of care. We also included studies that aimed to detect or quantify deviations from best practice across multiple components outlined by CPGs and CPs. Qualitative analyses (eg, interviews and ethnographic observations) that did not offer computer representations or measurable assessments of adherence were excluded. Additionally, studies that used a single process indicator as a proxy for adherence were excluded if they lacked a broader evaluation of best practice adherence.

Articles were excluded if they were not in English, had no full text available, or were not aligned with the research questions. Given the emerging nature of this topic and the evolving technological landscape, conference proceedings were also considered to capture developments in the field.

#### Study Selection

The retrieved articles were initially screened by applying the eligibility criteria by one reviewer (MTH). Concurrently, 5% of the total articles were randomly selected and separately screened by a second reviewer (MS). Discrepancies were discussed and resolved through consensus. The study selection process is illustrated in the PRISMA (Preferred Reporting Items for Systematic Reviews and Meta-Analyses) flow diagram.

### Data Charting and Data Items

A structured data charting form was developed to extract the following variables from each included study:

1. Bibliographic information (title, authors, year of publication, and publisher)
2. Study aims
3. Representation of clinical data
4. Representation of best practice standards
5. Whether deviations from clinical pathways were detected and measured
6. The mechanism used to measure adherence
7. Reported limitations

Full extraction details are available in Multimedia Appendix 1 [20,26-48].

#### Synthesis of the Result

Extracted data were analyzed using a narrative synthesis approach. Studies were grouped thematically based on their technical approaches to best practice representation and adherence measurement. Key features, methodological innovations, and limitations were summarized across studies. All authors contributed to iterative discussions, the refinement of the thematic structure, and the interpretation and presentation of the results.

## Results

### Included Articles and Metadata

**Figure 1.**
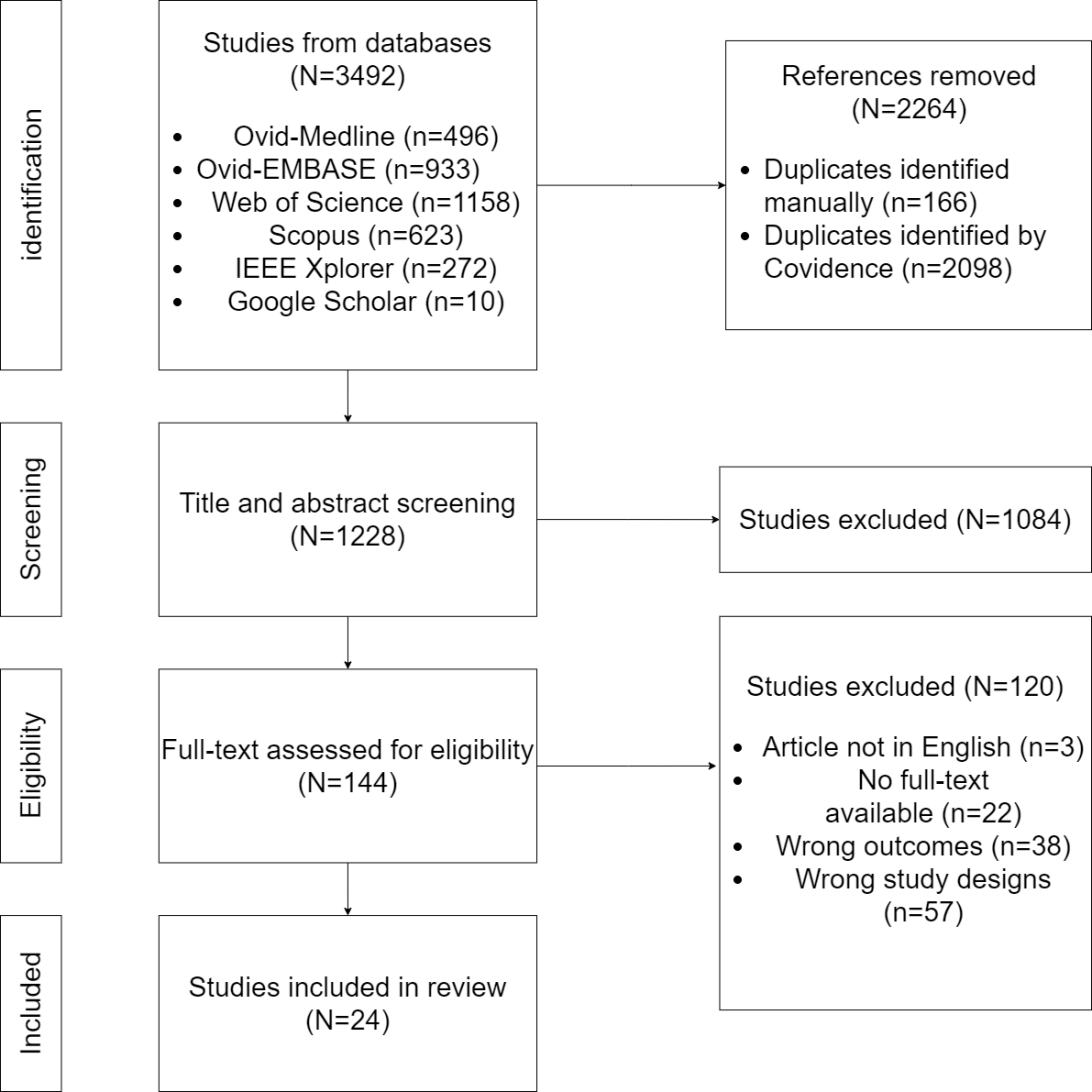
PRISMA (Preferred Reporting Items for Systematic Reviews and Meta-Analyses) flow diagram demonstrates the article search and selection process.

**Figure 2.**
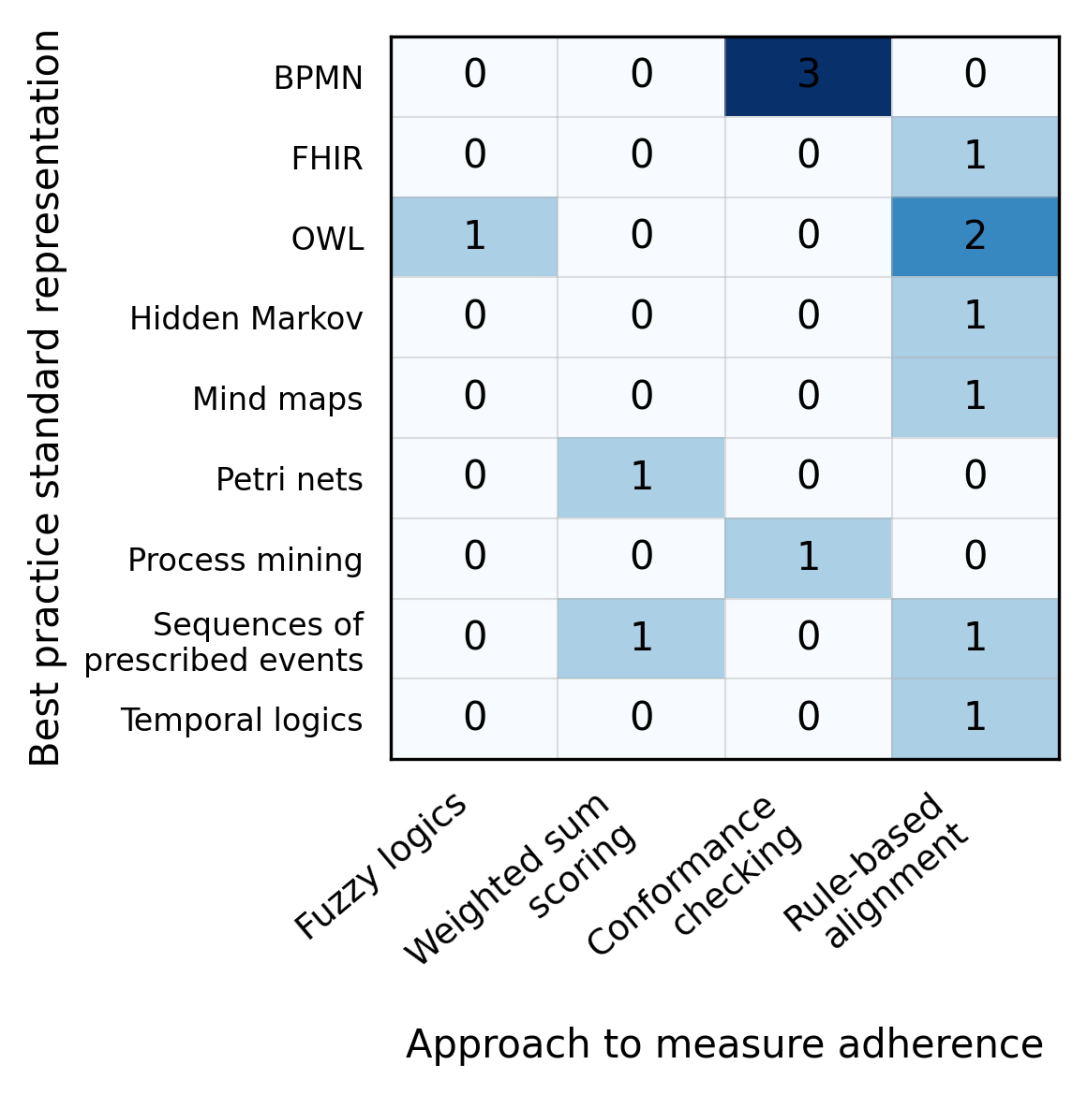
Map of best practice standards representation by approach to measure adherence in the included literature (N=14). BPMN: Business Process Model and Notation; FHIR: Fast Healthcare Interoperability Resources; OWL: Semantic Web Ontology Language.

For this scoping review, 24 articles [20,26-48] were included for data extraction (Figure 1). Most studies were conducted in China (6/24, 25%), the United States (5/24, 21%), and Germany (3/24, 13%). Of these, more than half (13/24, 54%) were published as conference proceedings rather than full-length journal articles, likely reflecting the technically innovative but emerging nature of this research area. Of 24 studies [20,26-48], 14 (58%) [20,26-36,46,48] included a measure of adherence to best practice standards (Figure 2). The number indicates how frequently each combination was used in the included studies. Formal knowledge representations—such as BPMN and Semantic Web Ontology Language (OWL)—combined with rule-based alignment are the most commonly used approach for representing best practice standards and measuring adherence.

### Clinical Conditions

Among the 24 included studies [20,26-48], only 1 study did not provide any clinical setting [26]. Cardiovascular disease was the most common clinical area, accounting for 54% (13/24) of the sample. Two studies applied their models across multiple clinical conditions (Table 1).

**Table 1.** Clinical conditions whose clinical practice guidelines or clinical pathways were translated to computer-interpretable guidelines in the included studies (N=24).

Clinical condition	Count
Weaning protocol in Intensive Care Unit (ICU)	1
Diabetes	1
Major joint replacement	1
Nutrition	1
Thyroid disease	1
No clinical case	1
COVID-19	2
Family planning and sexually transmitted infections	1
Cardiovascular disease	13
Mixed clinical conditions	2

#### Data Sources Used for Monitoring Adherence to Best Practice Standards

Various sources of clinical data were used in the included studies, including Health Level Seven (HL7) messages, structured clinical data extracted from eMR [27,28], patient events trace derived by sorting timestamped entries from eMR [20,29-31], and system-generated event logs [32-35]. Some studies transformed eMR data into standardized formats using the FHIR specification [36-38]. Conversely, several studies focused solely on the conceptual representation of best practice standards without implementation [26,39-46].

#### Representation of Best Practice Standards

Of the 24 studies [20,26-48], 13 [27,28,32,36,38-42,44-47] used CPGs as their reference for best practice, while the remaining studies used CPs. Various methods were used to transform CPGs and CPs to CIG format. The most used approaches were BPMN (6/24, 25%) and FHIR (4/24, 16%). For example, Yan et al [35] used BPMN to model a task-time matrix CP for unstable angina pectoris. The authors mapped terminology from eMR to pathway activities, identified key activities as milestones, and then embedded the remaining activities between those milestones to construct the process [35]. Parallel and inclusive gateways were used to represent concurrent activities, while exclusive gateways captured alternate clinical routes [35]. This BPMN representation yielded a computer-interpretable pathway model that served as a reference standard for detecting deviations in real-world care delivery [35]. Meanwhile, Lichtner et al [45] represented CPGs in FHIR by identifying the required elements and metadata from the CPGs to be captured in FHIR representation. An information model was built by organizing these elements into 3 main categories—medical relationships, metadata, and justification for the recommendations—and sorting them into 3 layers of certainty of evidence (primary clinical studies, systematic review and outcome-level assessment, and final recommendation derivation with evidence grading) [45]. Finally, the model was mapped into EBM-on-FHIR and CPG-on-FHIR profiles using resources such as PlanDefinition, EvidenceVariable, and ArtifactAssessment, thus enabling a computer-interpretable representation of CPG recommendations with underlying evidence certainty [45]. Details of the common methods used to represent CIGs are presented in Table 2.

**Table 2.** Description of the most used approaches to represent computer-interpretable guidelines.

Approach name	Included studies	Mechanism
Business Process Model and Notation (BPMN)	[32,33,35,37,44]	Map variables extracted from eMR^a^ to those components of CPGs^b^ and CPs^c^ Identify key activities in CPGs and CPs to act as milestones that connect different phases of the care process Add other care activities in between key activities Activities are sequenced based on the clinical logic: whether they occur simultaneously or exclusively, and the order in which activities occur first
Ontology (OWL)	[26,27,30,43,44]	Clinical concepts are defined as classes A clinical concept can have object properties which describe the relationship between clinical concepts and data properties which record its quantitative aspect eMR variables are then mapped to the newly created ontology to create instances of classes Decision rules are created based on the if-then logic to encompass the conditions as outlined in CPGs and CPs
FHIR	[36,37,40,45]	Identify the contents and metadata of CPGs and CPs to be captured in the FHIR-based^d^ representation Information model is built to understand the clinical concepts and relationships between them Components of CPGs and CPs are mapped to the corresponding FHIR resources Specifically, the PlanDefinition resource is used to model the care processes as outlined in CPGs and CPs. Meanwhile, the ActivityDefinition resource represents each care activity within the whole care process

a eMR: electronic medical record.

b CPG: clinical practice guideline.

c CP: clinical pathway.

d FHIR: Fast Healthcare Interoperability Resources.

Interestingly, some studies used hybrid approaches to represent best practice standards. For example, Dos Santos Leandro et al [37] used BPMN and FHIR to model stroke care CPs, first structuring the care process with BPMN, then mapping the components to corresponding FHIR resources to enable semantic interoperability. Whereas 2 studies [27,47] defined their best practice standards as linear sequences of prescribed actions without using formal modeling tools.

In addition to these core approaches, other approaches used to transform CPGs and CPs to CIGs included Petri Net [29], a combination of Natural Rule Language (NRL) and Object Constraint Language (OCL) [47], fuzzy logics [38], process mining [34], decision tree [28,39], hidden Markov model [31], and the formal representation language PROforma [41,42]. These studies thus demonstrate the use of a diverse range of formalisms to represent best practice standards; however, some of them lacked clinical integration, as observed in studies such as [39,41,42].

### Approaches to Measuring Adherence

The included studies used a range of techniques to detect and quantify deviations from best practices standards. The most commonly used approach involved rule-based alignment, wherein individual patient records were assessed against predefined care steps derived from CPGs and CPs [20,27-29,36,46,48]. In this approach, CPG and CP components are translated into computable “Event-Condition-Action” (ECA) constraints, each constraint representing a specific care step. Individual patient records were then systematically aligned with these predefined constraints using structured data extracted from eMRs. For example, Kaur et al [27] translated the nutrition recommendations into “rules” that dictate actions based on specific conditions or criteria using logical expressions or attributes (day of life, birth weight, and current health conditions). Similarly, Lichtner et al [36] used guideline recommendations in a FHIR-based declarative format and built executable ECA rules based on the criteria specified in the recommendations and then executed against standardized patient data to determine whether the patients’ data aligned with the recommendations. This rule-based alignment enables automated, transparent, and scalable assessment of adherence to best practice standards across large patient cohorts.

Beyond rule-based alignment, studies [20,29,48] incorporated weighting mechanisms to reflect the clinical significance of different types of deviations. Instead of treating all deviations from best practice standards as equally significant, these weighted scoring systems distinguished between addition, omission, and substitution of care activities and assigned differential weights. Implementation typically involves differentiating between types of activities that mismatch the prescribed activities in CPGs and CPs and those observed in patient data. In this context, omissions refer to activities specified in CPGs and CPs but not observed in the patient event trace, while additions refer to activities observed in the patient event trace that are not prescribed in CPGs and CPs.

By using weighting mechanisms, the adherence score reflects not only alignment with the referenced CPGs and CPs but also the severity of deviations. For example, van de Klundert et al [20] developed and tested dynamic programming formulations to match patient trajectories to the prescribed CPs, allowing partial adherence and assigning varying penalties to deviations based on their severity. Konrad et al [48] not only assigned equal weights to the addition and omission of activities compared to what was prescribed in the CP, but also to whether patient outcomes remained within target ranges during specified time intervals. Meanwhile, Zema et al [29] did not consider weighting patient outcomes, but they emphasized documentation quality in their Clinical Pathway Deviation Index (CPDI). CPDI was the weighted sum of 5 indicators: addition, omission, variation in activities sequence and duration, and missing in documentation. While the least weight was assigned to missing in documentation, equivalent weights were assigned to the other indicators.

Process mining techniques were used in a subset of studies to reconstruct care processes from event logs or event traces. Authors in several studies [29,30,32] used conformance checking and an optimal alignment algorithm to detect deviations. Deviations were identified by misalignment between observed activities and expected pathways, with some studies applying A* based algorithm—a graph traversal and path-finding algorithm—to determine the shortest or most efficient match between the observed and expected activities.

Unlike traditional health service research, which relies on single-point indicators, these studies adopted a process-level alignment approach. Rather than evaluating isolated components of CPGs and CPs, they aimed to align entire sequences of care activities with components extracted from best practice standards. This approach captures both what and when actions were performed, thus reflecting the temporal flow of care delivery. For example, Yan et al [26] described the modeling of the relative temporal relations between 2 clinical activities. They used temporal rules expressed in the Semantic Web Rule Language (SWRL) to describe the 30-minute interval between administering intravenous antibiotics and the cesarean delivery treatment intervention, as shown in Textbox 1 [26]. By analyzing these sequences, researchers can capture deviations in timing and sequencing, providing a more holistic and dynamic assessment of adherence to best practice standards.

Textbox 1. **Example of modeling the relative temporal relations between clinical activities using a Semantic Web Ontology Language (OWL).**

*begins(IVPuchAntibiotics, ?t1)* ^

*begins(CesareanDelivery, ?t2) ^ swrlb:*

*subtractDateTimesYieldingDayTimeDuration(?t, ?t2, ?t1)*

⇒*swrlb:greaterThan (?t,T30M)*.

Despite these methodological innovations, most studies focused on structural or process-level compliance and did not consider contextual factors such as patient conditions, comorbidities, or clinician intent. As a result, the distinction between warranted deviations and unwarranted deviations remained largely unresolved across the included studies.

## Discussion

### Principal Findings

This scoping review explored existing literature on the computable representations of best practice standards and methods for detecting and quantifying deviations from best practice using data extracted from eMRs. We identified 24 studies [20,26-48], a majority of which were published as conference proceedings. Cardiovascular disease was the most common clinical domain, most likely due to the extensive evidence available of CPGs in this clinical specialty [49].

A wide variety of methods were used to represent patient data, including system-generated event logs, patient event traces, and FHIR. Similarly, CIG models were constructed using BPMN, ontology frameworks, FHIR, or hybrid combinations of these. These models aim to enable structured and machine-readable, potentially interoperable standard-based guideline representation exchange format [36].

While not all studies detailed explicit methods for measuring adherence, the most common approach was rule-based alignment, wherein individual patient records were assessed against predefined care steps derived from CPGs and CPs. Some studies incorporated weighting mechanisms to differentiate the clinical significance of deviations, reflecting the recognition that not all deviations from recommendations are negative.

Three principal insights were identified. First, a diverse range of formalism types was used to represent best practice standards, though few were implemented in a clinically integrated fashion. Second, approaches to measuring adherence varied significantly, ranging from rule-based alignment to more nuanced methods involving weighted scoring and process mining. Finally, the effectiveness of adherence measurement was closely linked to the quality and structure of the underlying data sources, which varied across studies.

Together, these findings demonstrate both the progress made and the challenges that remain in operationalizing and measuring adherence to best practice using eMR-derived data. The results of this study highlight the need for standardized, context-sensitive, and interoperable systems that can support meaningful adherence monitoring to best practice standards as well as more quality and peer-reviewed research in the area.

### Representing Best Practice: Challenges in Formalization

Translating narrative CPGs and CPs into structured, computer-interpretable formats is inherently complex. The narrative language used in CPGs is often characterized by ambiguity, conditional logic, and implicit assumptions, making it difficult to convert into electronic formats without loss of fidelity. Many studies in this review used hierarchical decomposition of narrative text into modular components combined with temporal logic and decision rules to form the basis of CIGs. However, even when the logic is accurately encoded, clinicians may find it challenging to validate outputs from these systems as CIG languages are not always accessible for clinicians [50].

Moreover, the global implementation of these systems is often constrained by their design. Many of them were developed as standalone tools that do not communicate with each other or with existing health information systems [51]. Additionally, these systems are also highly tailored to specific use cases and cannot be easily modified or maintained, except by their original authors, making them unsustainable for broader adoption [17,52,53].

BPMN has the advantage of being a graphical representation that can visualize workflow processes, as demonstrated in the included studies (Table 2). In addition, a systematic review has also reinforced BPMN’s potential to standardize and redesign clinical processes [54]. However, there are certain limitations to using BPMN to represent best practice standards. Despite being excellent at modeling workflow, BPMN is primarily designed to model generic business processes and may not be able to capture the complexity and dynamics of health care processes [55,56]. Implementing BPMN in modeling clinical pathways requires a considerable investment in finance, time, and human resources [57]. At the same time, its technical complexity also poses a barrier to adoption among clinicians, especially those without formal training in informatics.

These limitations emphasize the need for models that balance clinical expressiveness with technical interoperability and usability, ensuring sustainability, scalability, and the ability to provide meaningful insights into clinical practice.

### Measuring Adherence: Methodological Tensions

Approaches to measuring adherence to best practice standards varied significantly, reflecting a wide methodological spectrum. Traditional approaches to measuring adherence to best practice standards often rely on external, aggregated metrics, such as length of stay, cost, infection rate, mortality, etc. Although these indicators can provide valuable insights at a system level, they offer little information about the underlying process of care, as well as patient-level information [58]. Such measures are insufficient to determine whether care was delivered in accordance with the sequence, timing, and logic prescribed by best practice standards.

Several studies in this review sought to overcome this barrier by aligning process-level components with guideline-specified components and patient data. These approaches thus provide a more granular view of whether the intended sequence and logic of care delivery were followed. Despite their methodological sophistication, most of these models did not integrate contextual data such as patient comorbidities, preferences, or clinical urgency.

This gap presents persistent tension. While current models are effective at detecting technical deviation, they remain limited in their ability to distinguish between clinically necessary deviations from those that are undesirable. The future approach to adherence measurement must evolve to be more patient-centered and context-aware.

### Limitations of Process Mining and Data Source Fidelity

Clinical decision-making is primarily guided by a patient’s health status and context, which are often outside the stated space that the traditional process mining techniques rely on [59]. Many process mining techniques can tell us which treatment behaviors are performed and in which order in accordance with established best practice standards [60]. These methods are attractive due to their scalability and ability to visualize workflow variation. However, their utility in clinical settings is often limited by the eMR data structure and granularity [61].

The effectiveness of process mining techniques is often hindered by the inconsistent, incomplete, or unstructured nature of eMR data. A recent scoping systematic review shows that process mining cannot distinguish the differences in deviations due to the complex nature of clinical data [62]. In addition, the authors mentioned that process mining becomes more beneficial as the input data become more structured, which is usually not the case, especially for data extracted from eMR [62].

Consequently, the accuracy of deviation detection in process mining is heavily dependent on data fidelity. Even when the result produced by process mining is technically significant, it may lack clinical relevance to clinicians.

Since clinical decision-making is primarily driven by patient-specific factors, many of which are not captured in structured eMR fields, process mining risks oversimplifying the complex care delivery process. Without integration of outcome data, clinician notes, or patient-specific modifiers, these methods offer only a partial view of adherence and may not resonate with clinical end users.

### Recent Approach Using FHIR for Interoperability and Real-Time Monitoring of Adherence to Best Practice Standards

Among the most promising recent developments in this domain is the adoption of FHIR. As an emerging interoperability standard, FHIR aims to advance the messaging standard to enhance the exchange and interpretation of information across disparate and heterogeneous systems [63]. In the included studies, FHIR was used to represent patient data in a format that supports automated adherence analysis. Therefore, the use of FHIR not only provides concise structural data input but also promotes the reusability of clinical data for research and accelerates data availability.

A recent scoping review, conducted by Tabari et al [64], provided a summary of the tools and techniques used to represent the FHIR-based data model, demonstrating the promising use of FHIR to facilitate the integration, transformation, and analysis of clinical data. Indeed, in this scoping review, as shown in Table 2, FHIR was used to represent best practice standards rather than solely represent patient data. FHIR was demonstrated to successfully translate guideline recommendations into a standardized, computer-interpretable format, coupled with a rating system that can assess the level of certainty of evidence [45]. The authors also performed a study on automating the integration of guideline representation with real-time clinical data. They found a promising possibility of real-time monitoring adherence to guideline recommendations [36]. In addition, a recent publication demonstrates that it is possible to set up a trustworthy process to develop computer-interpretable representations of quality indicators for future automated quality management systems [65]. As a result, advancements in adopting FHIR to monitor adherence to best practice standards demonstrate the possibility of a real-time adherence monitoring system that not only detects deviations but also weights them according to the certainty of the supporting evidence.

### Future Approach to Move Toward a More Meaningful Evaluation of Appropriate Care Delivery

Given the limitations of eMR data in measuring adherence to best practice standards, further research should aim to improve the applicability of eMR data for this purpose. This could be addressed by assessing the quality of eMR data characteristics—accuracy, completeness, reliability, relevance, and timelines—before evaluating adherence to CPGs and CPs. Furthermore, it could be beneficial to investigate the use of large language model technologies for the representation of CPGs and CPs and the measurement of adherence to best practice standards.

Despite these methodological innovations, most studies primarily focus on structural adherence, that is, whether the documented care sequence matched the prescribed components of CPGs and CPs, rather than on whether it was clinically appropriate in the specific care context. Consequently, while deviations from best practice standards can be detected with high accuracy, the crucial distinction between warranted deviations, which reflect high-quality patient-centered care, and unwarranted deviations, which can result in patient harm and unnecessary use of health care resources, remained largely unresolved.

Future studies can address this limitation by accounting for patient characteristics, outcomes, and clinician intent. For instance, detected deviations could be reclassified based on patient-specific conditions, allowing for outcome-informed validation of adherence measurement. By combining rule-based alignment and contextual reasoning, future approaches can advance beyond static measurement of adherence to best practice standards toward a more meaningful evaluation of appropriate care delivery. Additionally, future research should consider other potential factors influencing adherence to best practice standards, such as the structure of medical billing items, which could lead to deviations from the CPGs and CPs.

This scoping review makes several novel contributions to the existing literature by providing a comprehensive mapping of the computable representation of best practice standards and how adherence is measured—2 domains that have often been examined separately. Unlike previous reviews that focus only on either the technical approach or clinical evaluation, this scoping review synthesizes evidence from both perspectives and demonstrates how computer-interpretable format representation enables automated adherence assessment. It thus establishes a conceptual link between the modeling framework used for computable representation and the analytical methods used for evaluating practice variation. Notably, the findings promote the shift toward dynamic, computerized real-time monitoring of best practice standards, which can serve as a foundation for health care systems aiming to leverage eMR data for quality improvement. This also highlights the potential for a scalable, transparent, and clinically meaningful system for monitoring best practices to reduce unwarranted variation, strengthen guideline implementation, and ultimately improve patient care.

### Limitations

This scoping review has some limitations, including the restriction to English-language publications and databases indexed up to November 2025, which may have excluded relevant literature, especially from non–English-speaking countries. We also did not register this study protocol on PROSPERO. In addition, due to the heterogeneity of study designs, a formal quality appraisal was not performed. We also focused on the quantitative measurement of adherence to best practice standards. Other works had been done on investigating the deviations from best practice using qualitative approaches, which were not included and analyzed in this scoping review. However, the qualitative type of studies often focus on providing insights into the nature of deviations, as demonstrated in the studies done by Kahol et al [66], which may not fit well with our aims, as our study focuses on quantitative measurements. We also explored studies that proposed conceptual models rather than real-world implementation, limiting the generalizability of findings. Finally, we sourced studies with data extracted from eMR without considering additional data sources which again could provide the context of guideline deviations.

### Conclusions

This scoping review offers an innovative contribution by surveying and synthesizing the existing literature that highlights the significant progress made in the formalization of best practice standards and the development of quantitative measures for adherence monitoring. However, significant gaps remain. The representation of best practice standards is heterogeneous and often disconnected from clinical workflows. Adherence measurement tends to prioritize detectability over clinical relevance. Although various data sources have been leveraged for these approaches, many remain incomplete or underused.

The increasing adoption of FHIR highlights the potential to improve eMR-based adherence measurement; however, technical and operational barriers must be addressed to achieve these goals. To realize the full potential of eMR-based adherence monitoring, future work must focus on clinically meaningful modeling, standardized yet flexible adherence metrics, and real-time, interoperable systems that integrate seamlessly with care delivery. Such advances will be critical to supporting evidence-based, adaptive, and patient-centered health care systems.

## Supplementary material

10.2196/79937 Multimedia Appendix 1 Detailed properties of included studies.

10.2196/79937 Checklist 1 PRISMA checklist for scoping review.

10.2196/79937 Checklist 2 PRISMA-S checklist search strategies and search strings.

## Abbreviations

BPMN: Business Process Model and Notation
CDSS: Clinical Decision Support Systems
CIG: computer-interpretable guideline
CP: clinical pathway
CPDI: Clinical Pathway Deviation Index
CPG: clinical practice guideline
CQL: Clinical Quality Language
ECA: Event-Condition-Action
eMR: electronic medical record
FHIR: Fast Healthcare Interoperability Resources
HL7: Health Level Seven
NRL: Natural Rule Language
OCL: Object Constraint Language
OWL: Semantic Web Ontology Language
PRISMA: Preferred Reporting Items for Systematic Reviews and Meta-Analyses
PRISMA-S: An Extension of the Preferred Reporting Items for Systematic Reviews and Meta-Analyses Statement
PRISMA-ScR: Preferred Reporting Items for Systematic Reviews and Meta-Analyses Extension for Scoping Reviews
SWRL: Semantic Web Rule Language
