# Supplementary material for: Data-Driven Guideline Adherence in Data Representation and Compliance Measurement: Scoping Review
Source: J Med Internet Res. 2026 Feb 9;28:e79937. doi: 10.2196/79937 (PMC12885449; doi:10.2196/79937)
Supplement: Multimedia Appendix 1 [file jmir-v28-e79937-s001.docx]

**Detailed properties of articles included in the final synthesis.**

| Study ID | Title | Authors | Pub. Year | Journal or Proceedings | Country of Study | Clinical conditions | Sample size | Best practice standards | Representation of patient data | Type of EMR data | Representation of best practice standards | Compliance measure? | Deviation rate | Primary deviation reason | Mechanism of compliance measurement |
| --- | --- | --- | --- | --- | --- | --- | --- | --- | --- | --- | --- | --- | --- | --- | --- |
| 1 | CPDI: An Index for measuring deviations in Clinical Pathways | Zema M, et al | 2015 | 37th Annual International Conference of the IEEE Engineering in Medicine and Biology Society (EMBC) | Italy | a mix of clinical conditions | 24 | CP | event trace | structured | petri nets | yes | a CPDI for all PTs that was equal to 0.070 ± 0.029  (mean value ± standard deviation. it ranged  from 0.033 to 0.114, meaning that no PTs perfectly reflected  the corresponding CP (no PTs with CPDI equal to 0). | N/A | The CPDI index is the weighted sum of five indicators: addition, omission, missing documentation, variations in the sequence of activities, and duration. |
| 2 | A model-driven approach to clinical practice guidelines representation and evaluation using standards | Farkash A, Timm J, Waks Z. | 2013 | Studies in Health Technology and Informatics. MEDINFO 2013 | US, Israel | cardiovascular disease-related | N/A | CPG | patient event extracted from HL7 Continuity of Care Document | N/A | Natural Rule Language and Object Constraint Language | no | N/A | N/A | N/A |
| 3 | A model-driven transformation approach for the modelling of processes in clinical practice guidelines | Marta­nez-Salvador B. et al | 2023 | Artificial Intelligence in Medicine. | Spain | cardiovascular disease-related | N/A | CPG | N/A | N/A | BPMN and PROforma using ATLAS Transformation Language (ATL) | no | N/A | N/A | N/A |
| 4 | Enhancing narrative clinical guidance with computer-readable artifacts: Authoring FHIR implementation guides based on WHO recommendations | Shivers J. et al. | 2021 | Journal of Biomedical Informatics | US, Switzerland | family planning & sexually transmitted infections | N/A | CPG | N/A | N/A | FHIR | no | N/A | N/A | N/A |
| 5 | Representation of evidence-based clinical practice guideline recommendations on FHIR | Lichtner G. et al | 2023 | Journal of Biomedical Informatics | Germany | COVID-19 | N/A | CPG | N/A | N/A | FHIR | no | N/A | N/A | N/A |
| 6 | FHIR Implementation Guide for Stroke: A dual focus on the patient’s clinical pathway and value-based healthcare | dos Santos Leandro et al | 2024 | International Journal of Medical Informatics | Brazil | cardiovascular disease-related | N/A | CP | FHIR | N/A | BPMN + FHIR | no | N/A | N/A | N/A |
| 7 | A semantics-based clinical pathway workflow and variance management framework | Yan Ye, Jiang Zhibin | 2008 | 2008 IEEE International Conference on Service Operations and Logistics, and Informatics | China | no clinical case | N/A | CP | N/A | N/A | OWL and Protégé | yes | N/A | N/A | fuzzy logics + petri nets |
| 8 | Automated monitoring of adherence to evidence-based clinical guideline recommendations: design and implementation study | Lichtner et al. | 2023 | Journal of Medical Internet Research | Germany | COVID-19 | 2104 | CPG | FHIR | Structured | FHIR | yes | 70% of relevant patients were treated according to the recommendation | A large proportion of patients were treated with steroids according to guideline recommendation before arriving at the intensive care unit of the hospital | Alignment recommendations generated rules with patients' data. |
| 9 | Measuring clinical pathway adherence | van de Klundert J. | 2010 | Journal of Biomedical Informatics | The Netherland | cardiovascular disease-related | 12103 in total from 2001 to 2005 | CP | event trace | Structured | sequences of prescribed events | Yes | The average 5^th^ decile of  Aneurysm: 0.64  Stroke: 0.53  Heart failure:0.6  Ischemic heart disease: 0.46 | N/A | Dynamic programming formulations to match patient trajectories to the prescribed clinical pathways |
| 10 | Use of mind maps and iterative decision trees to develop a guideline-based clinical decision support system for routine surgical practice: case study in thyroid nodules | Yu et al | 2019 | Journal of the American Medical Informatics Association | Korea | thyroid disease | 483 | CPG | EMR data | Structured | mind map + iterative decision tree | yes | 21.1% discordant rate | Either an unexpected histological finding during intraoperative frozen biopsy lymph node analysis, or a refusal on the part of the patient to undergo total thyroidectomy | Alignment recommendations generated rules with patients' data. |
| 11 | Variance analysis in task-time matrix clinical pathways | Yan H. et al | 2017 | 2017 IEEE EMBS International Conference on Biomedical & Health Informatics (BHI) | China | cardiovascular disease-related | 1051 | CP | event logs | Structured | BPMN | yes | The deviation ratio median occurred at 30-39%, which is defined as the number of deviations divided by the length of the optimal alignment | Perform ECG was done out of the original order, and Give Drugs Relating to Myocardial Ischemia Protection are not specified in the CP | conformance checking using a* algorithm for optimal alignment and process mining |
| 12 | An Interpretable Guideline Model to Handle Incomplete Information | Oliverira T. et al | 2012 | Distributed Computing and Artificial Intelligence: 9th International Conference | Portugal | cardiovascular disease-related | N/A | CPG | N/A | N/A | PROforma, incorporates a quality of information evaluation method using Extended Logic Programming (ELP) | no | N/A | N/A | N/A |
| 13 | Signal from the noise: A mixed graphical and quantitative process mining approach to evaluate care pathways applied to emergency stroke care | Noshad M, Rose C., Chen J | 2014 | Journal of Biomedical Informatics | US | cardiovascular disease-related | 269 | CP | event logs | structured | mixed graphical + unsupervised quantitative process mining. | yes | Conformity score 0.36 (0.2 – 0.64) | The included patients were managed from out-of-hospital stroke activation, so their ordering and timing of events deviate from the most common path | conformity score calculation by mapping the time and probability from a summarized vector of nodes (events) and edges (events occurring in series) |
| 14 | Fuzzy on FHIR: a Decision Support service for Healthcare Applications | Minutolo A. | 2016 | International Conference on P2P, Parallel, Grid, Cloud Internet Computing | Italy | cardiovascular disease-related | N/A | CPG | FHIR | structured | fuzzy logics | no | N/A | N/A | N/A |
| 15 | An Ontology and Rule-Based Clinical Decision Support System for Personalized Nutrition Recommendations in the Neonatal Intensive Care Unit | Kaur R. et al | 2023 | IEEE Access | US | nutrition | 601 | CPG | EMR data | structured | OWL used to create ontologies Protégé | yes | 48% compliant with Nutrition Recommendation Ontologies recommendations in patients without major morbidities | N/A | Alignment recommendations generated rules with patients' data. |
| 16 | Supporting Clinical Guidelines Using DL-Temporal Reasoning | Autexier S. et al | 2013 | 2013 10th International Conference and Expo on Emerging Technologies for a Smarter World (CEWIT) | Germany | cardiovascular disease-related | N/A | CPG | N/A | N/A | Description and temporal logics | yes | N/A | N/A | Alignment recommendations generated rules with patients' data. |
| 17 | Modeling of Clinical Pathways based on Ontology | Zhen H. et al | 2009 | 2009 IEEE International Symposium on IT in Medicine & Education | China | a mix of clinical conditions | N/A | CP | N/A | N/A | OWL | No | N/A | N/A | N/A |
| 18 | Online Treatment Compliance Checking for Clinical Pathways | Zhengxing Huang Â· Yurong Bao Â· Wei Dong Â· Xudong Lu Â· Huilong Duan | 2014 | Journal of Medical Systems | China | cardiovascular disease-related | N/A | CP | event trace | Structured | OWL | yes | N/A | N/A | rule-based CP compliance checking system |
| 19 | Monitoring adherence to evidence-based practices: A method to utilize HL7 messages from hospital information systems | R. Konrad1; B. Tulu1; M. Lawley | 2013 | Applied Clinical Informatics | US | major joint replacement | 17 | CP | patient event extracted from HL7 messages | structured | sequences of prescribed events | yes | N/A | N/A | Weighted sum scoring of addition. Omission activities, and monitoring patients’ outcomes |
| 20 | An Ontological Clinical Decision Support System Based on Clinical Guidelines for Diabetes Patients in Sri Lanka | Madhusanka S. et.al | 2020 | Healthcare | Sri Lanka | diabetes | N/A | CPG | N/A | N/A | BPMN + OWL | no | N/A | N/A | N/A |
| 21 | Building a Knowledge-Based Tool for Auto-Assessing the Cardiovascular Risk | UGON A. et al | 2018 | Studies in Health Technology and Informatics | France | cardiovascular disease-related | 15 | CPG | N/A | N/A | Decision tree | no | N/A | N/A | N/A |
| 22 | Evaluating the relative value of care interventions based on clinical pathway variation detection and propensity score | Xu H. et al | 2020 | 2020 IEEE International Conference on Bioinformatics and Biomedicine (BIBM) | China | cardiovascular disease-related | 3276 | CPG | event logs |  | BPMN | Yes | N/A | Neuroprotective agent (48894 times) is the most common deviation detected | conformance checking using a* algorithm for optimal alignment and process mining |
| 23 | On accurate, automated and insightful deviation analysis of clinical protocols | Yan H | 2018 | 2018 IEEE International Conference on Bioinformatics and Biomedicine (BIBM) | The Netherland | Weaning protocol in ICU | 65 | CP | event logs | structured | BPMN | yes | N/A | N/A | conformance checking using a* algorithm for optimal alignment.+ process mining |
| 24 | Automatic Variance Analysis of Multistage Care Pathways | Li X et al | 2014 | Studies in Health Technology and Informatics | China | cardiovascular disease-related | 430 | CP | event trace | structured | hidden Markov model | yes | From stage A to stage C, the absent activities rate is 44%, 27%, and 4.1%, respectively, while the violated constraints rate is 54%, 50%, and 53%, respectively. | Non-compliance with the pre-defined lab tests. Many indications planned in the CP were not monitored on schedule. | First-order linear temporal logic and binomial tests on three categories of activities: addition, omission, and constraint-violated. |
