# Supplementary material for: Data-Driven Guideline Adherence in Data Representation and Compliance Measurement: Scoping Review
Source: J Med Internet Res. 2026 Feb 9;28:e79937. doi: 10.2196/79937 (PMC12885449; doi:10.2196/79937)
Supplement: Checklist 2 [file jmir-v28-e79937-s003.docx]

**Search Strategy according to PRISMA-S Checklist and Search Strings of Scoping Review in Each Database**

1. **PRISMA-S Checklist**

| **Section/topic** | **#** | **Checklist item** | **Location(s) Reported** |
| --- | --- | --- | --- |
| **INFORMATION SOURCES AND METHODS** | | | |
| Database name | 1 | Name each individual database searched, stating the platform for each. | Line 136-137 |
| Multi-database searching | 2 | If databases were searched simultaneously on a single platform, state the name of the platform, listing all of the databases searched. | Line 136 - 137 |
| Study registries | 3 | List any study registries searched. | Line 137 |
| Online resources and browsing | 4 | Describe any online or print source purposefully searched or browsed (e.g., tables of contents, print conference proceedings, web sites), and how this was done. | Line 137-138 |
| Citation searching | 5 | Indicate whether cited references or citing references were examined, and describe any methods used for locating cited/citing references (e.g., browsing reference lists, using a citation index, setting up email alerts for references citing included studies). | Line 145-146 |
| Contacts | 6 | Indicate whether additional studies or data were sought by contacting authors, experts, manufacturers, or others. | Line 146-147 |
| Other methods | 7 | Describe any additional information sources or search methods used. | Line 145-147 |
| **SEARCH STRATEGIES** | | | |
| Full search strategies | 8 | Include the search strategies for each database and information source, copied and pasted exactly as run. | Appendix B |
| Limits and restrictions | 9 | Specify that no limits were used, or describe any limits or restrictions applied to a search (e.g., date or time period, language, study design) and provide justification for their use. | Line 142-143 |
| Search filters | 10 | Indicate whether published search filters were used (as originally designed or modified), and if so, cite the filter(s) used. | Line 138-142 |
| Prior work | 11 | Indicate when search strategies from other literature reviews were adapted or reused for a substantive part or all of the search, citing the previous review(s). | Line 138-142 |
| Updates | 12 | Report the methods used to update the search(es) (e.g., rerunning searches, email alerts). | Line 138 |
| Dates of searches | 13 | For each search strategy, provide the date when the last search occurred. | Line 138 |
| **PEER REVIEW** | | | |
| Peer review | 14 | Describe any search peer review process. | Line 138-140 |
| **MANAGING RECORDS** | | | |
| Total Records | 15 | Document the total number of records identified from each database and other information sources. | Figure 1 line 180-181 |
| Deduplication | 16 | Describe the processes and any software used to deduplicate records from multiple database searches and other information sources. | Line 148-151 |
|  |  |  |  |
| PRISMA-S: An Extension to the PRISMA Statement for Reporting Literature Searches in Systematic Reviews | | |  |
| Rethlefsen ML, Kirtley S, Waffenschmidt S, Ayala AP, Moher D, Page MJ, Koffel JB, PRISMA-S Group. | | |  |
| Last updated February 27, 2020. | |  |  |

1. **Search strings in each database**
2. **Ovid MEDLINE(R)**

1 (guideline* or protocol* or recommendation* or "best practice" or "clinical pathway").mp

2 ((provider* or clinician* or nurse* or physician* or doctor* or practitioner* or practice) adj5 (deviation* or variation* or divergene* or discrepane* or "non-comlian*" or noncomplian* or "non-adheren*" or nonadheren* or variance)).mp.

3 1 and 2

4 (score* or measure* or account* or calculate* or index* or count* or quantify* or represent* or monitor* or algorithm* or utility*).mp.

5 (electronic medical record* or electronic health record* or electronic patient record* or EMR or EHR or "patient information system" or "longitudinal patient data" or "hospital information system" or "patient data").mp.

6 (clinical decision support system or computer-assisted decision making or computer-assisted diagnosis or clinical decision-making or clinical alarm* or clinical alert* or clinical decision rules or rule-based system or CDSS or CDS).mp.

7 5 or 6

8 3 and 4 and 7

1. **Embase Classic + Embase**

1 (guideline* or protocol* or recommendation* or "best practice" or "clinical pathway").mp

2 ((provider* or clinician* or nurse* or physician* or doctor* or practitioner* or practice) adj5 (deviation* or variation* or divergene* or discrepane* or "non-comlian*" or noncomplian* or "non-adheren*" or nonadheren* or variance)).mp.

3 1 and 2

4 (score* or measure* or account* or calculate* or index* or count* or quantify* or represent* or monitor* or algorithm* or utility*).mp.

5 (electronic medical record* or electronic health record* or electronic patient record* or EMR or EHR or "patient information system" or "longitudinal patient data" or "hospital information system" or "patient data").mp.

6 (clinical decision support system or computer-assisted decision making or computer-assisted diagnosis or clinical decision-making or clinical alarm* or clinical alert* or clinical decision rules or rule-based system or CDSS or CDS).mp.

7 5 or 6

8 3 and 4 and 7

1. **Scopus**

( TITLE-ABS-KEY ( ( guideline* OR protocol* OR recommendation* OR "best practice" OR "clinical pathway" ) AND ( ( provider* OR clinician* OR nurse* OR physician* OR doctor* OR practitioner* OR practice ) W/5 ( deviation* OR variation* OR divergene* OR discrepane* OR "non-comlian*" OR noncomplian* OR "non-adheren*" OR nonadheren* OR variance ) ) ) AND TITLE-ABS-KEY ( score* OR measure* OR account* OR calculate* OR index* OR count* OR quantify* OR represent* OR monitor* OR algorithm* OR utility* ) AND TITLE-ABS-KEY ( ( electronic AND medical AND record* OR electronic AND health AND record* OR electronic AND patient AND record* OR emr OR ehr OR "patient information system" OR "longitudinal patient data" OR "hospital information system" OR "patient data" ) OR ( clinical AND decision AND support AND system OR computer-assisted AND decision AND making OR computer-assisted AND diagnosis OR clinical AND decision-making OR clinical AND alarm* OR clinical AND alert* OR clinical AND decision AND rules OR rule-based AND system OR cdss OR cds ) ) )**IV. IEEE**

(((electronic medical record OR patient longitudinal data OR hospital information system OR clinical decision making OR clinical decision support)) AND (score OR measure OR account OR calculate OR index OR count OR quantify OR represent OR monitor OR algorithm)) AND ((All Metadata:guideline* OR All Metadata:protocol* OR All Metadata:best practice OR All Metadata:clinical pathway) AND (All Metadata:deviat* OR All Metadata:variant* OR All Metadata:variance OR All Metadata:divergenc* OR All Metadata:discrepan* OR All Metadata:non-compliance OR All Metadata:non-adherence))

1. **Web of Science**

1: (TI=((guideline* or protocol* or recommendation* or “best practice” or “clinical pathway”))) OR AB=((guideline* or protocol* or recommendation* or “best practice” or “clinical pathway”)) OR AK=((guideline* or protocol* or recommendation* or “best practice” or “clinical pathway”)) OR KP=((guideline* or protocol* or recommendation* or “best practice” or “clinical pathway”)) OR TS=((guideline* or protocol* or recommendation* or “best practice” or “clinical pathway”))

2: (((TI=(((provider* or clinician* or physician* or doctor* or nurse* or practice or practitioner*) NEAR (deviation* or variation* or divergenc* or discrepanc* or “non-complian*” or noncomplian* or “non-adheren*” or nonadheren*)))) OR AB=(((provider* or clinician* or physician* or doctor* or nurse* or practice or practitioner*) NEAR (deviation* or variation* or divergenc* or discrepanc* or “non-complian*” or noncomplian* or “non-adheren*” or nonadheren*)))) OR AK=(((provider* or clinician* or physician* or doctor* or nurse* or practice or practitioner*) NEAR (deviation* or variation* or divergenc* or discrepanc* or “non-complian*” or noncomplian* or “non-adheren*” or nonadheren*)))) OR KP=(((provider* or clinician* or physician* or doctor* or nurse* or practice or practitioner*) NEAR (deviation* or variation* or divergenc* or discrepanc* or “non-complian*” or noncomplian* or “non-adheren*” or nonadheren*))) OR TS=(((provider* or clinician* or physician* or doctor* or nurse* or practice or practitioner*) NEAR (deviation* or variation* or divergenc* or discrepanc* or “non-complian*” or noncomplian* or “non-adheren*” or nonadheren*)))

3: (((TI=(score* or measure* or account* or calculate* or index* or count* or quantify* or represent* or monitor* or algorithm* or utility*)) OR (AB=(score* or measure* or account* or calculate* or index* or count* or quantify* or represent* or monitor* or algorithm* or utility*)) OR (AK=(score* or measure* or account* or calculate* or index* or count* or quantify* or represent* or monitor* or algorithm* or utility*)) OR (KP=(score* or measure* or account* or calculate* or index* or count* or quantify* or represent* or monitor* or algorithm* or utility*)) OR (TS=(score* or measure* or account* or calculate* or index* or count* or quantify* or represent* or monitor* or algorithm* or utility*))))

4: (((TI=(electronic medical record* or electronic health record* or electronic patient record* or EMR or EHR or "patient information system" or "longitudinal patient data" or "hospital information system" or "patient data")) OR AB=(electronic medical record* or electronic health record* or electronic patient record* or EMR or EHR or "patient information system" or "longitudinal patient data" or "hospital information system" or "patient data")) OR AK=(electronic medical record* or electronic health record* or electronic patient record* or EMR or EHR or "patient information system" or "longitudinal patient data" or "hospital information system" or "patient data")) OR KP=(electronic medical record* or electronic health record* or electronic patient record* or EMR or EHR or "patient information system" or "longitudinal patient data" or "hospital information system" or "patient data") OR TS==(electronic medical record* or electronic health record* or electronic patient record* or EMR or EHR or "patient information system" or "longitudinal patient data" or "hospital information system" or "patient data")

5: (((TI=(clinical decision support system or computer-assisted decision making or computer-assisted diagnosis or clinical decision-making or clinical alarm* or clinical alert* or clinical decision rules or rule-based system or CDSS or CDS)) OR AB=(clinical decision support system or computer-assisted decision making or computer-assisted diagnosis or clinical decision-making or clinical alarm* or clinical alert* or clinical decision rules or rule-based system or CDSS or CDS)) OR AK=(clinical decision support system or computer-assisted decision making or computer-assisted diagnosis or clinical decision-making or clinical alarm* or clinical alert* or clinical decision rules or rule-based system or CDSS or CDS)) OR KP=(clinical decision support system or computer-assisted decision making or computer-assisted diagnosis or clinical decision-making or clinical alarm* or clinical alert* or clinical decision rules or rule-based system or CDSS or CDS) OR TS==(clinical decision support system or computer-assisted decision making or computer-assisted diagnosis or clinical decision-making or clinical alarm* or clinical alert* or clinical decision rules or rule-based system or CDSS or CDS)

6: #1 AND #2

7: #5 OR #4

8: #6 AND #7 AND #3

1. **IEEE Xplore**

[((analyze OR score OR measure OR account OR calculate OR index OR quantify OR represent OR monitor OR algorithm OR utility))) AND ((All Metadata:guideline* OR All Metadata:protocol* OR All Metadata:"best practice" OR All Metadata:"clinical pathway") AND (All Metadata:deviat* OR All Metadata:variant* OR All Metadata:variance OR All Metadata:divergenc* OR All Metadata:discrepan* OR All Metadata:"non-compliance" OR All Metadata:"non-adherence"))) AND (electronic medical record OR patient longitudinal data OR hospital information system OR clinical decision making OR clinical decision support)](https://ieeexplore.ieee.org/search/searchresult.jsp?contentType=all&sortType=&filter=-ContentType+EQ+%22Newsletters%22&rowsPerPage=100&searchField=Search_All&combineQuery=sortType%3D%26filter%3D-ContentType+EQ+%22Newsletters%22%26searchField%3DSearch_All.OPAND.filter%3D-ContentType+EQ+%22Newsletters%22%26searchField%3DSearch_All%26queryText%3D%28analyze+OR+score+OR+measure+OR+account+OR+calculate+OR+index+OR+quantify+OR+represent+OR+monitor+OR+algorithm+OR+utility%29.OPAND.filter%3D-ContentType+EQ+%22Newsletters%22%26matchBoolean%3Dtrue%26searchField%3DSearch_All%26queryText%3D%28Search_All%3Aguideline*+OR+Search_All%3Aprotocol*+OR+Search_All%3A%22best+practice%22+OR+Search_All%3A%22clinical+pathway%22%29+AND+%28Search_All%3Adeviat*+OR+Search_All%3Avariant*+OR+Search_All%3Avariance+OR+Search_All%3Adivergenc*+OR+Search_All%3Adiscrepan*+OR+Search_All%3A%22non-compliance%22+OR+Search_All%3A%22non-adherence%22%29.OPAND.filter%3D-ContentType+EQ+%22Newsletters%22%26searchField%3DSearch_All%26queryText%3Delectronic+medical+record+OR+patient+longitudinal+data+OR+hospital+information+system+OR+clinical+decision+making+OR+clinical+decision+support&history=no)
